# May’s Diseases of Women

**Published:** 1890-09

**Authors:** 


MAY'S DISEASES OF WOMEN. Being a Concise and Systematic Exposition of the Theory and Practice of Gynecology. For the use of Students and Practitioners. Second edition, revised by LEONARD S. RAU, M. D., Attending Physician to Harlem Hospital, Out-patient Department, New York, etc., with thirty-one illustrations on wood. Small 8vo; pp. 373. Philadelphia: Lea Brothers & Co. 1890.

This volume is a compilation of the teachings of various American, German, and English authors, which the author says he has "condensed, classified, and arranged, to make the book a short and systematic treatise on the theories and practice of the diseases peculiar to women, which shall represent the accepted views in this modern science." It is also further declared to be its purpose to aid the student who has perused larger works in his review of the subject.

If this kind of literature is desirable, then this book ought to meet the demand, for viewed from this standpoint the work must be pronounced well done. We have discovered nothing new in its pages to direct attention to, hence shall be content in simply stating the features and object of the book, and let each judge for himself as to the desirability of possessing it. It is well printed on good paper with plain type, and is substantially bound.

